# Protective Action of Neurotrophic Factors and Estrogen against Oxidative Stress-Mediated Neurodegeneration

**DOI:** 10.1155/2011/405194

**Published:** 2011-05-31

**Authors:** Tadahiro Numakawa, Tomoya Matsumoto, Yumiko Numakawa, Misty Richards, Shigeto Yamawaki, Hiroshi Kunugi

**Affiliations:** ^1^Department of Mental Disorder Research, National Institute of Neuroscience, National Center of Neurology and Psychiatry, Tokyo 187-8502, Japan; ^2^Core Research for Evolutional Science and Technology Program (CREST), Japan Science and Technology Agency (JST), Saitama 332-0012, Japan; ^3^Department of Psychiatry and Neurosciences, Division of Frontier Medical Science, Graduate School of Biomedical Sciences, Hiroshima University, 1-2-3 Kasumi, Minami-ku, Hiroshima 734-8551, Japan; ^4^Peptide-prima Co., Ltd., 1-25-81, Nuyamazu, Kumamoto 861-2102, Japan; ^5^The Center for Neuropharmacology and Neuroscience, Albany Medical College, Albany, NY 12208, USA

## Abstract

Oxidative stress is involved in the pathogenesis of neurodegenerative disorders such as Alzheimer's disease, Parkinson's disease, and Huntington's disease. Low levels of reactive oxygen species (ROS) and reactive nitrogen species (RNS) are important for maintenance of neuronal function, though elevated levels lead to neuronal cell death. A complex series of events including excitotoxicity, Ca^2+^ overload, and mitochondrial dysfunction contributes to oxidative stress-mediated neurodegeneration. As expected, many antioxidants like phytochemicals and vitamins are known to reduce oxidative toxicity. Additionally, growing evidence indicates that neurotrophic factors such as brain-derived neurotrophic factor (BDNF) and estrogens significantly prevent neuronal damage caused by oxidative stress. Here, we review and discuss recent studies addressing the protective mechanisms of neurotrophic factors and estrogen within this system.

## 1. Introduction

It is well established that the brain consumes a large quantity of oxygen and glucose [1–5]. Brain neurons utilize such nutrients, requiring a consistent and steady supply in order to function appropriately. Not surprisingly, brain neurons are vulnerable to oxidative stress [6], which threatens the overall functionality of the brain. Though various systems protecting against oxidative toxicity exist in the brain at cellular and molecular levels, a disruption of the defensive system may be involved in neurological deficits observed in neurodegenerative diseases. Indeed, many studies suggest that oxidative toxicity is related to Alzheimer's disease (AD), Parkinson's disease (PD), and Huntington's disease (HD) [7]. In addition, a correlation between an accumulation of oxidative stress and aging has also been established [8]. Thus, it is important to clarify the detailed relationship between oxidative stress and cellular damage in neurodegenerative diseases and the aging process. In the cellular and molecular mechanisms underlying oxidative stress-induced cell death, it is well known that excitotoxicity, Ca^2+^ overload, mitochondrial dysfunction, and the stimulation of intracellular signaling cascades play a role [9]. As expected, antioxidants including many phytochemicals and vitamins have been found to support the survival of neurons under oxidative stress. 

Brain-derived neurotrophic factor (BDNF), a member of the neurotrophin family, is known to be a strong survival-promoting factor against various neuronal insults. As a result, the molecular mechanisms underlying neurotrophin-dependent survival promotion when exposed to oxidative stress have been extensively studied. BDNF plays a critical role in cell proliferation, cell differentiation, neuronal protection, and the regulation of synaptic function in the central nervous system (CNS) via stimulating key intracellular signaling cascades [10, 11]. In addition to BDNF, glial cell line-derived neurotrophic factor (GDNF) and hepatocyte growth factor (HGF) are also effective for neuronal survival [12, 13]. Furthermore, estrogens, which regulate synaptic plasticity in addition to sex differentiation of the brain [14–16], are found to exert protective actions against toxic conditions such as oxidative stress [17]. Here, we review the current issues concerning protective functions of neurotrophic factors and estrogen on neurons under oxidative stress.

## 2. The Role of Oxidative Stress in Neurodegenerative Diseases

**Figure 1:**
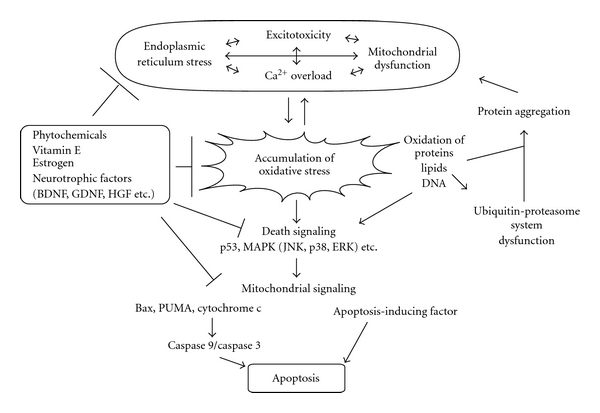
Mechanisms underlying oxidative stress-mediated neuronal apoptosis. Accumulation of oxidative stress is involved in the development/progression of neurodegenerative diseases. A number of events including excitotoxicity, mitochondrial dysfunction, Ca^2+^ overload, and endoplasmic reticulum stress are associated with excess reactive oxygen species (ROS) and reactive nitrogen species (RNS) generation. High levels of ROS/RNS lead to oxidation of proteins, lipids, and DNA. Oxidized lipids induce damage of the ubiquitin-proteasome system (UPS). The UPS dysfunction and oxidation of proteins result in aggregation of proteins, recognized as a hallmark of several neurodegenerative diseases. Under oxidative stress, death signaling pathways (p53, mitogen-activated protein kinase (MAPK), etc.) are activated. Activation of p53 leads to induction of proapoptotic proteins such as Bax and p53-upregulated modulator of apoptosis (PUMA), followed by translocation of these proteins into mitochondria. Finally, mitochondrial cytochrome c is released, which then stimulates the activation of caspase 9/caspase 3. Alternatively, mitochondria secrete apoptosis-inducing factor (AIF), leading to caspase-independent apoptosis. As shown, recent studies suggest antioxidant effects of phytochemicals, vitamin E, estrogen, and neurotrophic factors including brain-derived neurotrophic factor (BDNF), glial cell line-derived neurotrophic factor (GDNF), and hepatocyte growth factor (HGF), leading to increased preservation of neuronal function.

Low levels of ROS and RNS have a physiological effect on cellular functions including neuronal plasticity [18]. However, in excess, ROS/RNS cause oxidation/nitrosylation of lipids, proteins, and nucleic acids, resulting in neuronal cell death (Figure 1). Such damage occurs as a result of either overproduction of ROS/RNS or reduced activity of enzymatic and nonenzymatic antioxidants. Thus, the delicate balance between pro- and antioxidant reactions is critical for maintaining normal neuronal function. 

Oxidative stress-mediated toxicity may be closely related to the pathogenesis of neurodegenerative diseases such as AD, PD, and HD [7]. For example, in AD brains, markers for protein oxidation (protein carbonyls and 3-nitro-tyrosine (3-NT)), lipid oxidation (4-hydroxy-2′-nonenal (4-HNE)), and DNA oxidation (8-hydroxy-2-deoxyoguanine (8-OHdG)) are elevated [19]. Indeed, the accumulation of amyloid beta (A*β*), a hallmark of AD, produces ROS including hydrogen peroxide (H_2_O_2_) in the presence of Fe^3+^ or Cu^2+^ [20–22], but see [23]. In PD brains, in which a selective and progressive loss of dopamine (DA) neurons in the substantia nigra pars compacta occurs, 4-HNE, protein carbonyls, 3-NT, and 8-OHdG are all increased while glutathione (GSH, a major intracellular antioxidant) is decreased [24]. Interestingly, 4-HNE covalently binds to alpha-synuclein (*α*-Syn), a central protein in PD pathogenesis, resulting in neurotoxic effects on DAergic and GABAergic neuronal cultures [25]. Similarly, HD brains (where significant neuronal loss in the striatum and cortex is observed) demonstrate elevated 3-NT, lipofuscin (a product of unsaturated fatty acid peroxidation), malondialdehyde (a marker for lipid oxidation), and 8-OHdG [26]. Reduced levels of GSH were also confirmed in cultured neurons from mice expressing mutant Huntingtin protein (Htt140Q/140Q) [27]. 

Oxidative toxicity is also involved in cerebral ischemia/reperfusion injury. Brain regions and types of neurons that are vulnerable to ischemia are limited. It may be because cerebral blood flow is highly spatiotemporally modulated [2], and this view could also be important to understand why specific types of neurons in different brain regions are affected in each neurodegenerative disease. In addition, a large body of evidence suggests that accumulation of oxidative stress-dependent damage occurs during normal aging, which may cause a noticeable decline in cognitive function [8, 28]. Considering that cognitive deficits are observed in neurodegenerative diseases such as AD as well, a common mechanism underlying oxidative stress-mediated neuronal cell death may exist. In the following section, we summarize the current knowledge concerning oxidative stress-mediated neuronal cell death. 

## 3. Oxidative Stress-Mediated Neuronal Cell Death

### 3.1. Mitochondrial Dysfunction, Ca^2+^ Overload and Excitotoxicity

Apoptosis, a prototypic form of programmed cell death, is a major mode of cell death in neurodegenerative diseases. Various mechanisms including excitotoxicity, Ca^2+^ overload, mitochondrial dysfunction, endoplasmic reticulum stress, and oxidative stress have been found to contribute to apoptosis [9] (Figure 1). Mitochondria produce low levels of ROS in a process known as cellular respiration through the electron transport chain (ETC). The ETC consists of five protein complexes (I–V), and a disruption of this electron transport system leads to excess generation of ROS [29]. Importantly, a number of studies reported possible involvement of mitochondrial dysfunction, including altered activity of the ETC, in patients and animal models for AD [30], PD [31], HD [32], and stroke [33]. Some reports suggest that patients with psychiatric disorders, such as schizophrenia [34], depression [35], and bipolar disorder [36], also display mitochondrial dysfunction. 

In addition, mitochondria regulate/impact/affect Ca^2+^ homeostasis by sequestering excess cytosolic Ca^2+^ into their matrix (named Ca^2+^ loading). However, an uncontrolled Ca^2+^ loading may be involved in neurodegeneration. In a study investigating striatal mitochondria of Hdh150 knock-in HD mice, a disrupted Ca^2+^ homeostasis was found [37]. Another study discovered that a deficiency of phosphatase and tensin homolog deleted on chromosome 10 (PTEN)-induced putative kinase 1 (PINK1, a mitochondrial kinase linked to familial PD) results in mitochondrial Ca^2+^ accumulation in cultured neurons [38]. Endoplasmic reticulum also regulates intracellular Ca^2+^ concentration through inositol-1,4,5-triphosphate receptors (InsP3Rs) and ryanodine receptors (RyRs). Interestingly, presenilin (PS) 1 and 2, genes involved in the pathogenesis of AD, acted as a passive endoplasmic reticulum Ca^2+^ channel to maintain steady-state Ca^2+^ levels, which was disrupted by mutant PS1-M146V and PS2-N141I [39, 40]. These PS mutants enhanced the gating activity of InsP3Rs, leading to A*β* generation [41]. Furthermore, it was shown that A*β*-containing senile plaques cause Ca^2+^ overload [42]. Taken together, it seems likely that mutant PSs and A*β* contribute to the disruption of Ca^2+^ homeostasis, which may cause mitochondrial dysfunction leading to neuronal degeneration [30]. 

Remarkably, nicotinamide adenine dinucleotide phosphate (NADPH) oxidase (Nox) may generate ROS in a mitochondria-independent manner. In cultured cortical neurons lacking p47(phox), a cytosolic subunit of Nox, extensive N-methyl-D-aspartic acid (NMDA) receptor activation failed to produce ROS, while H_2_O_2_ or the mitochondrial complex III inhibitor (antimycin) increased ROS [43]. Furthermore, ROS production and oxidative damage in the hippocampal CA1 neurons after ischemia were dramatically attenuated in mice either treated with Nox inhibitor or lacking gp91(phox), another Nox subunit [44]. Considering the fact that overactivation of NMDA receptors occurs in ischemia [45], it is possible that NMDA-mediated excitotoxicity may cause mitochondria-independent, but Nox-dependent, ROS production in cerebral ischemia/reperfusion injury. 

### 3.2. Signaling Pathways in Apoptosis

p53, a transcription factor, is activated by ROS, and induces the upregulation of mitochondrial proapoptotic proteins including B-cell lymphoma-2-associated X protein (Bax) and members of the B-cell lymphoma-2-homology 3 (BH3) family consisting of BH3 interacting death agonist (Bid), Nox activator 1 (Noxa), and p53-upregulated modulator of apoptosis (PUMA) [33]. Indeed, oxidative stressors including H_2_O_2_ increased Noxa, Bim, and PUMA (but not Bid) in cultured cortical neurons [46]. Importantly, PUMA, but not Noxa or Bim, was involved in Bax-dependent apoptosis [46]. The contribution of p53/PUMA to delayed cell death of hippocampal neurons after stroke was also reported [47]. These studies suggest that p53-mediated PUMA expression may be a key event in neuronal apoptosis (Figure 1). 

As the final step of apoptosis, cytochrome c is released from mitochondria via the permeability transition pore (PTP), which consists of the mitochondrial inner and outer membrane proteins including B-cell lymphoma-2 (Bcl-2) and Bax (Figure 1). Cytosolic cytochrome c participates in the formation of the apoptosome, a multiprotein complex including apoptosis protease-activating factor 1 (Apaf-1) and caspase-9, which activates caspase-3, an executioner in cell death [48]. On the other hand, apoptosis-inducing factor (AIF) is involved in mitochondria-mediated, but caspase-independent, apoptosis [49] (Figure 1).

### 3.3. Antioxidative Factors

Considering that oxidative stress may be associated with the pathogenesis of neurodegenerative diseases, a key therapeutic intervention would be to block or delay accumulating oxidative stress levels via increasing the function of endogenous antioxidants and/or suppressing ROS production (Figure 1). Well-known antioxidants include glutathione precursor [50, 51], polyphenols [52–54], catechins [55], flavonoids [56], and sulfated polysaccharides [57]. As the toxicity of phytochemicals is low, these substances offer a new therapeutic approach against neurodegenerative diseases [58]. On the other hand, whether oxidative stress is a cause or consequence of neurodegenerative disease remains to be elucidated [7]. A growing body of evidence suggests that oxidative stress directly initiates and progresses to neuronal cell death. However, it is possible that accumulation of oxidative stress is easily induced in neurons weakened by other insults. Indeed, in the apoptotic process, many cellular events including mitochondrial dysfunction, Ca^2+^ overload, and excitotoxicity activate death signaling cascades (Figure 1). Such negative feedback loops may influence cell viability. These events probably occur in parallel and have an additive or synergic effect in the induction of cell death. Therefore, in addition to blocking accumulation of oxidative stress, inhibiting death-signaling cascades and activating survival signaling would also be effective. In the following section, we focus specifically on neurotrophic factors and steroid hormones that may exert a beneficial influence. 

## 4. Neurotrophins and Oxidative Stress in Neurodegenerative Diseases

As mentioned above, oxidative stress may be involved in the onset of HD, AD, PD, and amyotrophic lateral sclerosis (ALS) [7, 9]. Interestingly, neurotrophic factors, including neurotrophins, may also be associated with the pathology of these neurodegenerative diseases. For example, both mRNA and protein levels of BDNF are decreased in patients and animal models of HD [59–61]. In addition, the level of TrkB (tropomyosin-related kinase B), a high affinity receptor for BDNF, is also reduced in knockin HD striatal cells, in which mutant huntingtin with 111 glutamines (7 glutamines in normal) is expressed [62]. Following TrkB activation stimulated by BDNF, the mitogen-activated protein kinase/extracellular signal-regulated protein kinase (MAPK/ERK), phospholipase C*γ* (PLC*γ*), and phosphatidylinositol 3-kinase (PI3K) pathways are primarily triggered [10]. In the knock-in HD striatal cells, a down-regulation of ERK signaling occurred, while PI3K/Akt and PLC*γ* pathways were intact. Such a decrease in ERK signaling in these striatal cells resulted in an increase in the cell death caused by H_2_O_2_ [63]. As expected, it was revealed that BDNF, neurotrophin-3 (NT-3), and NT-4/5 prevent neuronal cell death in an animal model of HD [64]. 

Recent reports suggest that the upregulation of BDNF expression/function plays a role in neuroprotection within AD models. Counts and Mufson showed that noradrenaline (NA) is neuroprotective against A*β*-dependent toxicity in human NTera-2N (hNT) neurons and rat hippocampal neurons [65]. NA prevented an increase in ROS caused by A*β*. Notably, coapplication with functional blocking antibodies for BDNF or nerve growth factor (NGF) significantly inhibited the NA-dependent protective effect against A*β* toxicity [65]. As AD is well known as an age-related neurodegenerative illness, the senescence-accelerated mouse prone 8 (SAMP8) mice, which show age-related impairment of cognitive function, is a useful model of AD [66]. Using the SAMP8 mice, Zhao et al. investigated the effect of ginsenoside, a component of ginseng, on memory [67]. They reported that chronic treatment with ginsenoside prevented loss of memory in aged SAMP8 mice. Such a treatment with ginsenoside decreased the A*β* and, in turn, increased antioxidation and synaptic plasticity-related proteins such as BDNF [67]. 

Oxidative stress may damage nigral DA neurons, resulting in the onset of PD. Under oxidative stress, heme oxygenase-1 (HO-1) increases and exerts a positive effect on nigral DA neurons. Overexpression of HO-1 in rat substantia nigra rescued DA neurons from cell death caused by 1-methyl-4-phenylpyridinium (MPP(+)), which is an inhibitor for mitochondrial complex I and is well known to produce PD symptoms. After HO-1 overexpression, GDNF, in addition to BDNF, was upregulated [68]. Additionally, it was reported that bilirubin, a downstream product of HO-1, increased GDNF and BDNF expression through ERK and PI3K/Akt pathways [69]. These results suggest that HO-1 protects neurons through increasing these neurotrophic factors. A role of the novel DA D3 receptor agonist D-264 in neuroprotection was reported [70]. In the 1-methyl-4-phenyl-1,2,3,6-tetrahydropyridine (MPTP, an inhibitor of mitochondrial complex I)-induced neurodegeneration mouse model for PD, D-264 treatment improved behavioral performance and reduced neuronal loss. Remarkably, the D-264 treatment induced an upregulation of BDNF and GDNF in MPTP-treated animals [70]. Finally, using an in vitro system, L-theanine (a glutamate analog) was shown to promote SH-SY5Y cell survival and inhibited downregulation of both BDNF and GDNF under neurotoxicant (rotenone and dieldrin) application [71]. Generally, GDNF and BDNF are important for survival/morphological change of DA neurons, and both have a recovery effect on PD-like behavior [12, 72, 73]. Taken together, it is possible that upregulation of growth factors including BDNF and GDNF is necessary for the prevention of DA neuronal damage. 

## 5. BDNF and Oxidative Stress-Induced Cell Death

BDNF exerts protective effects against neuronal cell death by activating intracellular signaling cascades via TrkB [10, 11, 74]. Interestingly, trypanosome trans-sialidase (TS, sialic acid-transferring enzyme) mimics neurotrophins. Woronowicz et al. showed that TS induced phosphorylation of TrkB in rat pheochromocytoma (PC12) cells expressing TrkB and promoted cell survival under H_2_O_2_ stress [75]. The PI3K pathway was important for TS-mediated survival promotion. On the other hand, BDNF protects cultured cortical neurons from NMDA- or H_2_O_2_-induced cell death via suppressing the MAPK pathway [76]. Once exposed to NMDA or H_2_O_2_, retinoblastoma protein and E2F1 transcription factor, which are cell cycle regulators, were stimulated. BDNF inhibited such activation of cell cycle regulators, suggesting that the prevention of cell cycle reentry is involved in BDNF function during oxidative stress [76]. Moreover, the activation of cyclic adenosine 3′,5′-monophosphate (cAMP)-responsive element-binding protein (CREB) is involved in BDNF neuroprotection. Transgenic mice expressing A-CREB, a dominant negative form of CREB, showed a significant increase in vulnerability to seizure activity. The A-CREB mice demonstrated increased ROS levels and decreased neuroprotection by BDNF application, suggesting that CREB is an essential upstream effector of neuroprotection against oxidative toxicity [77]. Importantly, CREB also regulates the transcriptional production of BDNF [78]. The BDNF gene consists of nine exons, and exon IX corresponds to the common open reading frame of the protein. The remaining exons have distinct promoters, respectively. Thus, the transcript of BDNF consists of one of eight 5′ untranslated exons (exon I~VIII) and 3′ exon IX [79]. Interestingly, the action of CREB via promoter IV is critical for experience-dependent production of BDNF [80]. Therefore, positive-feedback mechanisms may be involved in BDNF-mediated neuroprotection. 

As mentioned, BDNF seems to be beneficial in the therapeutic approach to neurodegenerative diseases. However, previous clinical trials have revealed numerous side effects of neurotrophins as well as their poor penetration through the blood brain barrier, making it very difficult to use these proteins as a drug [81]. Therefore, many studies have been performed in an effort to find a drug that upregulates BDNF. In SH-SY5Y cells after H_2_O_2_ application, tripterygium regelii extract (TRE), a traditional herbal medicine, increased tyrosine hydroxylase, a dopaminergic marker, and BDNF [82]. TRE was shown to repress the upregulation of proapoptotic proteins Bax and caspase-3, while inhibiting downregulation of antiapoptotic Bcl-2 under H_2_O_2_ application [82]. Sonic hedgehog (SHH) protein, a member of the Hedgehog family of signaling molecules [83], is putatively involved as a neuroprotective agent in oxidative stress-related neurodegenerative disease and ischemia. After H_2_O_2_ exposure, the SHH pathway was stimulated in cultured cortical neurons, and the increase in SHH pathway activation was noticeably protective against cell death caused by H_2_O_2_ [84]. In that in vitro system, exogenous SHH increased levels of vascular endothelial growth factor (VEGF) and BDNF, as well as activity of superoxide dismutase (SOD) and Bcl-2 expression [84]. 

Positive effects of the antioxidant vitamin E on oxidative stress-mediated toxicity in vitro [85–87] and in vivo [88, 89] have been reported. Vitamin E has also been shown to exert beneficial effects against neurodegenerative diseases [90, 91]. Our research demonstrates that pretreatment with vitamin E analogs including *α*- and *γ*-tocopherol (*α*T and *γ*T, respectively) and *α*- and *γ*-tocotrienol (*α*T3 and *γ*T3) protected cultured cortical neurons against H_2_O_2_-mediated neuronal cell death [92]. In our cultures, *α*T stimulated the activation of both the ERK and PI3K pathways and caused the upregulation of Bcl-2. Importantly, *α*T-mediated survival and Bcl-2 upregulation disappeared in the presence of inhibitors for ERK and PI3K signaling, suggesting the involvement of both pathways in neuroprotection by vitamin E analogs. However, the neuroprotection was not via BDNF signaling, as *α*T unchanged TrkB activation and BDNF expression [92]. It would be interesting to examine possible contributions from other neurotrophic factors. 

It is now critical to further investigate the mechanisms underlying the upregulation of BDNF and/or other effective growth factors in order to discover more efficacious medications. In general, BDNF levels are regulated by neuronal activity. In addition to the influx of Ca^2+^, neuronal activity, including glutamatergic regulation, contributes to the production and secretion of BDNF [93–98]. Change in the production and secretion of BDNF is thought to be involved in the activity-dependent synaptic plasticity in the CNS [99, 100]. Interestingly, two recent studies have demonstrated the role of synaptic activity in neuroprotection. In cultured hippocampal neurons, action potential bursting reduced the levels of p53, PUMA and Apaf1 [101]. Furthermore, NMDA receptor stimulation inhibited PUMA-mediated apoptosis via reducing levels of Apaf1 and procaspase-9 [102]. In support of these current studies, a previous study demonstrated that transcranial magnetic stimulation, which is well known to potentiate neuronal activity, inhibited toxic effects of 3-nitropropionic acid (3-NPA) (protein/lipid oxidations, reduction in activities of catalase, GSH peroxidase and succinate dehydrogenase, and GSH deficiency) and rescued the striatal neuronal loss in rats [103]. It is necessary to investigate whether or not such neuronal activity-mediated protection occurs via the upregulation of BDNF. Additionally, future studies investigating the role of neuronal activity in the expression of neurotrophic factors that are influenced by molecules that cross the blood brain barrier are needed.

Transplantation of growth factor-secreting cells may serve as an alternative method to treat neurodegenerative diseases. Indeed, the grafting of neurotrophin-secreting cell lines has been shown to protect neurons against quinolinate-induced cell death in an animal model of HD [64]. In addition, it was shown that erythropoietin-transduced human mesenchymal stromal cells (EPO-MSCs) played a neuroprotective role in the rat model for ischemic stroke [104]. In the EPO-MSCs, neurotrophic factors including BDNF and HGF were upregulated. The implantation of EPO-MSCs into ischemic rats reversed impairment in neurological function and infarct volumes [104]. Finally, a gene transfer approach may be a potentially effective strategy as well. In an in vivo cognitive dysfunction model induced by A*β* injection, HGF gene transfer improved impairment of cognitive behavior. It was suggested that BDNF upregulation was involved in the positive action of HGF gene transfer [105]. Further investigation on the possible mechanisms underlying the BDNF upregulation is interesting. 

## 6. Estrogen Signaling and Oxidative Stress

Estrogen, one of the sex steroids, has various roles in sex differentiation, neuroprotection, and synaptic plasticity [14–16, 106]. Furthermore, estrogenic protection from toxicity including excitotoxicity and oxidative stress is well studied [107–109]. Importantly, the maintenance of mitochondrial function is linked to estrogenic protection under toxic stress. Protein phosphatases influence activation levels of kinase signaling and of mitochondrial apoptosis-related proteins, and such intracellular mechanisms are closely associated with estrogenic protection [110]. 

Generally, estrogens are believed to regulate transcription of target genes via estrogen receptor *α* (ER*α*) and ER*β*. Estrogens bind to ER*α* and ER*β*, exerting various effects via initiating diverse intracellular signaling cascades. Specifically, the discovery of ER*β* prompted major developments leading towards the understanding of estrogenic function [111, 112]. In addition, it has been recently suggested that estrogens also exert their effects via ER-mediated nongenomic or non-ER-mediated functions. 

Estrogens protect neurons from severe conditions including oxidative stress. 17*β*-estradiol (E2), one of the estrogens, reduces CA1 hippocampal cell death following global cerebral ischemia [113]. In that in vivo system, Nox activity and superoxide production in the hippocampal region were repressed by E2 application. Interestingly, extranuclear ER*α*-dependent nongenomic function, including the activation of Akt, is involved in the E2 effect [113]. Xia et al. examined the effect of selective ER ligands on glutamate toxicity. In cultured cortical neurons, R,R-tetrahydrochrysene (R,R-THC, ER*β* antagonist and ER*α* agonist) displays a neuroprotective effect against glutamate-induced cell death [114], suggesting an important role of ER*α* in estrogen-mediated neuroprotection. On the other hand, a knockdown of ER*β* induced a lower resting mitochondrial membrane potential in immortal hippocampal and primary hippocampal neurons [115]. The ER*β* knockdown resulted in maintenance of adenosine 5′-triphosphate (ATP) concentration, and decreased mitochondrial superoxide levels under H_2_O_2_ stress. As expected, the neuronal loss of ER*β* knockdown cells diminished in the presence of oxidative stress caused by glutamate or H_2_O_2_ [115]. Recently, the novel function of GPR30, a G protein-coupled ER, has been reported. Gingerich et al. found that pretreatment with E2 decreased cell death caused by glutamate, which may be partially mediated by GPR30 [116]. 

It is possible that ER*β* regulates neuronal activity. As a result of neurotransmission, spontaneous Ca^2+^ oscillations occured and our group previously showed potentiation in glutamate-mediated Ca^2+^ oscillation after BDNF addition [117]. In our cortical cultures, voltage-dependent Ca^2+^ channels and ionotropic glutamate receptors contributed to the spontaneous Ca^2+^ oscillations, and BDNF-induced glutamate release was critical for the potentiation in the oscillations. Recently, Zhang et al. found that selective ER*β* agonists (not ER*α* agonists) rapidly potentiated Ca^2+^ oscillations in neurons derived from embryonic stem cells and activated protein kinase C (PKC), Akt, and ERK pathways. Interestingly, nifedipine, a blocker of L-type voltage-dependent Ca^2+^ channels, abolished these ER*β* actions [118], suggesting that estrogen regulates neuronal function via ER*β*. Remarkably, membrane-localized ER*α* activates mGluR5 signaling (one of the metabotropic glutamate receptors) to stimulate CREB in striatal neurons. Furthermore, both ER*α* and ER*β* activate mGluR3 to attenuate L-type voltage-dependent Ca^2+^ channel-mediated CREB activation [119]. Considering that CREB is involved in the transcriptional production of BDNF [78], the action of these ERs may affect BDNF levels in neuronal cells. 

## 7. Estrogen and Ca^2+^ Homeostasis under Oxidative Stress

**Figure 2:**
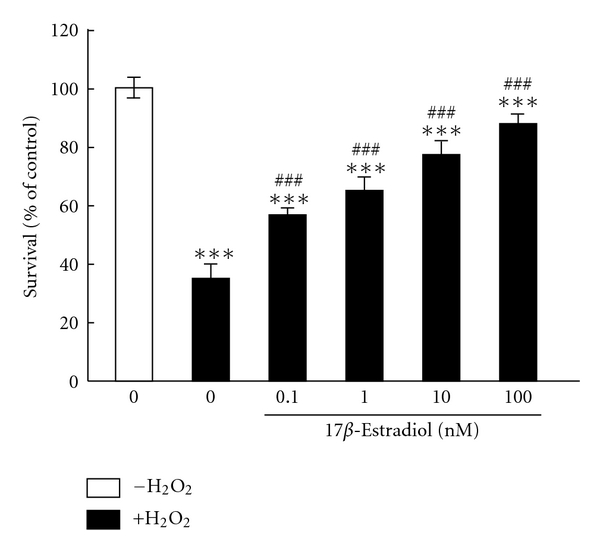
17*β*-estradiol prevents cortical neurons from cell death caused by H_2_O_2_ exposure. Dissociated cortical neurons were prepared from cerebral cortex of postnatal 2-day-old rats. At 6 days in vitro, 17*β*-estradiol was applied at indicated concentrations. Twenty-four hours later, H_2_O_2_ (final 50 *μ*M) was added to induce cell death. Following an additional twelve-hour culture, cell survival was determined using an MTT (tetrazolium salt) assay. Data represent mean ± S.D. (*n* = 6). ***P < .001 versus control (no H_2_O_2_). ^###^ *P* < .001 versus no estradiol + H_2_O_2_.

**Figure 3:**
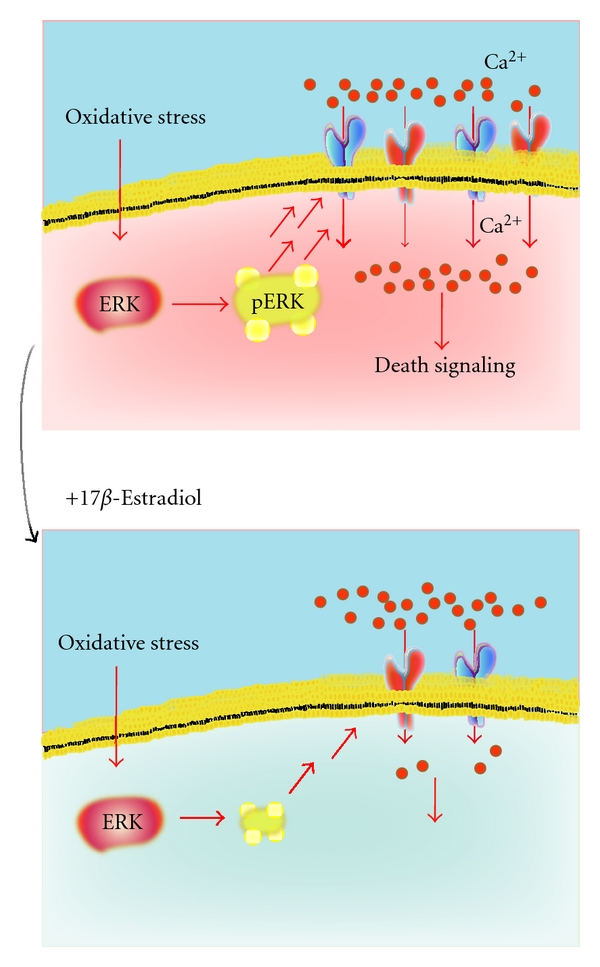
17*β*-estradiol inhibits neuronal cell death under oxidative stress via reducing the series of events evoked by exposure to H_2_O_2_, including overactivation of the ERK signaling and overload of Ca^2+^. *Upper*: After H_2_O_2_ addition, marked phosphorylated (activated) ERK (pERK) and resultant increase in intracellular Ca^2+^ concentration were observed, resulting in cell death. *Lower*: Pretreatment with 17*β*-estradiol induced downregulation of ionotropic glutamate receptors via decreasing ERK activation, while also serving to decrease levels of Ca^2+^ influx triggered by H_2_O_2_. Such a decrease in glutamate receptor expression and intracellular Ca^2+^ was also confirmed in the presence of U0126, an inhibitor of ERK signaling. As expected, chronic 17*β*-estradiol reduced levels of pERK stimulated by H_2_O_2_. A blockade of glutamate receptors rescued cortical cells from H_2_O_2_-dependent death. Therefore, it is possible that 17*β*-estradiol promotes survival via suppressing glutamate receptor-mediated Ca^2+^ influx, due to downregulation of ionotropic glutamate receptors [120].

Using cultured cortical neurons, we demonstrated the protective effect of E2 against cell death under oxidative stress caused by H_2_O_2_ [120] (Figure 2). Members of the MAPK family including c-jun N-terminal kinase (JNK) [121], p38 [122], and ERK [123, 124] play pivotal roles in neuronal apoptosis [125] (Figure 1). In our system, the exposure to H_2_O_2_ triggered the overactivation of the ERK pathway, leading to an abnormal increase in intracellular Ca^2+^ concentration (Figure 3). In general, perturbations of Ca^2+^ homeostasis are related to apoptosis in various cell populations [126–131]. In our neurons, the abnormal Ca^2+^ accumulation caused by H_2_O_2_ was significantly decreased by E2 pretreatment, or in the presence of U0126, an inhibitor for ERK signaling [120]. Recently, we reported that ERK signaling plays a role in maintaining adequate expression levels of glutamate receptors [132–134]. Importantly, chronic E2 treatment induced the downregulation of ionotropic glutamate receptor subunits including NR2A and GluR2/3. Such a decrease in glutamate receptor expression levels was also confirmed after U0126 addition. Indeed, such E2 treatment suppressed the overactivation of ERK pathway stimulated by H_2_O_2_. Furthermore, inhibitors of ionotropic glutamate receptors blocked cell death caused by H_2_O_2_. Taken together, it is possible that E2 exerts survival-promoting effects through repressing glutamate receptor-mediated Ca^2+^ influx [120] (Figure 3). As described, ERK signaling is essential for maintenance of glutamate receptor levels, making it interesting to investigate how estrogens influence ERK signaling. 

p66^Shc^ also generates mitochondrial ROS (H_2_O_2_), causes impairment in Ca^2+^ homeostasis, and is associated with apoptosis [135, 136]. Almeida et al. found that H_2_O_2_ stimulates PKC*β*/p66^Shc^/NF-*κ*B signaling to apoptosis in osteoblastic cells, and that E2 prevents H_2_O_2_-dependent activation of p66^Shc^ and NF-*κ*B via repressing phosphorylation of PKC*β*, resulting in protection from cell death [137]. 

## 8. Estrogen In Vivo Approach

In 6-hydroxydopamine (6-OHDA, a PD mimetic)-lesioned rats, a neuroprotective effect of silymarin (SM, one of flavonolignans) was shown [138]. SM administration protected neurons of the substantia nigra pars compacta from 6-OHDA toxicity, while fulvestrant, an ER antagonist, partially blocked the effects of SM. Additionally, the effect of oral estrogen on ROS generation was reported. Wing et al. demonstrated a beneficial effect of chronic oral estrogen treatment on oxidative stress and atherosclerosis in apoE-deficient mice [139]. Using ovariectomized apoE-deficient mice, it was revealed that atherosclerosis was reduced when treated with E2 (6 *μ*g/day) for 12 weeks. Importantly, after E2 treatment, superoxide anion and expression of Nox decreased, while Cu/ZnSOD and MnSOD increased [139]. Last, Schwann cells (SC) play a critical role in spinal cord injury repair, though SC survival after transplantation is very difficult. Current research is focused on discovering if E2 pretreatment protects SC, in an effort to generate more successful spinal cord transplantation procedures [140]. In primary SC cultures, strong expression of ER*α* and ER*β*, and overall E2-dependent survival mechanisms against H_2_O_2_ exposure were confirmed, though ICI182780 (an ER antagonist) had no influence on E2 effects. These findings suggest that genomic signaling via ERs is not involved. Importantly, in spinal cord injury, sustained E2 administration was found to be an effective treatment improving SC transplantation [140]. 

## 9. Conclusion

An increase in neuronal damage at the cellular and molecular level may be involved in the pathogenesis of brain illness, including neurodegenerative disease. It is possible that oxidative stress leads to neuronal cell death via increasing glutamate-mediated excitotoxicity, intracellular Ca^2+^ concentration, mitochondrial dysfunction, activation of death-signaling cascades, and decreasing overall survival signaling. Several drug candidates, which were found to attenuate deleterious symptoms in various models of neurodegenerative disease, are reported to upregulate the expression of neurotrophic factors including BDNF. Considering this, it seems pertinent to further investigate the possible mechanisms underlying such neurotrophic factor upregulation. On the other hand, estrogenic survival promotion is also well studied, though further investigation addressing how each ER contributes to neuronal protection against oxidative toxicity is needed. Finally, as a close relationship between steroid hormones and BDNF in various neuronal functions including cell survival is known [141], detailed studies concerning how estrogen and BDNF interact with each other in CNS neurons under oxidative stress are important.
